# An Autopsy of Nanofiltration Membrane Used for Landfill Leachate Treatment

**DOI:** 10.1155/2015/850530

**Published:** 2015-06-02

**Authors:** Ibrahim Demir, Ismail Koyuncu, Serkan Guclu, Senol Yildiz, Vahit Balahorli, Suphi Caglar, Turker Turken, Mehmet E. Pasaoglu, Recep Kaya, Reyhan Sengur-Tasdemir

**Affiliations:** ^1^Environmental Engineering Department, Istanbul Technical University, Maslak, 34469 Istanbul, Turkey; ^2^National Research Center on Membrane Technologies (MEM-TEK), Istanbul Technical University, Maslak, 34469 Istanbul, Turkey; ^3^ISTAC, Istanbul Environmental Management in Industry and Trade Inc., 34379 Istanbul, Turkey; ^4^Nanoscience and Nanoengineering Department, Istanbul Technical University, Maslak, 34469 Istanbul, Turkey

## Abstract

Komurcuoda leachate treatment plant, Istanbul, which consists of membrane bioreactor (MBR) and nanofiltration (NF) system, faced rapid flux decline in membranes after 3-year successful operation. To compensate rapid flux decline in membranes, the fouled membranes were renewed but replacement of the membranes did not solve the problem. To find the reasons and make a comprehensive analysis, membrane autopsy was performed. Visual and physical inspection of the modules and some instrumental analysis were conducted for membrane autopsy. Membranes were found severely fouled with organic and inorganic foulants. Main foulant was iron which was deposited on surface. The main reason was found to be the changing of aerator type of MBR. When surface aerators were exchanged with bottom diffusers which led to increasing of dissolved oxygen (DO) level of the basin, iron particles were oxidized and they converted into particulate insoluble form. It was thought that probably this insoluble form of the iron particles was the main cause of decreased membrane performance. After the diagnosis, a new pretreatment alternative including a new iron antiscalant was suggested and system performance has been recovered.

## 1. Introduction

Membrane processes have many advantages due to their modular designs, small footprints, and automated operations [1]. These advantages increased their usage within very wide range of applications in water and wastewater treatment. Despite this wide usage, membrane lifetime limits the sustainability of the membrane processes. Widespread use of membranes is still restricted because of membrane fouling phenomena [2]. Membrane lifetime and performance are affected by many factors such as selection of proper process membrane material and operating parameters. Membrane performance losses can be recognized by low effluent rates, decreased rejection, high pressure drop between inlet and outlet, and frequent cleaning requirement [3]. Optimum operation conditions decrease replacement period and operating costs and increase membrane performance. Another option to increase the lifetime of the membrane is to apply pretreatment [4]. However, fouling is one of the biggest problems in membrane area which negatively effects membrane performance. Membrane fouling control is an important subject and effective fouling control can be verified by membrane autopsy [5].

Renewal of membranes can be due to membrane clogging or complete lifetime. Membrane autopsy may help to extend membrane lifetime. Membrane autopsy can be thought of as starting point for problem determination. It also gives information about optimum operating conditions, chemical cleaning effectiveness, and the effects of influent water characteristics on membrane performance. According to the studies, generally membranes encounter biofouling, metal oxide fouling, oxidation, abrasion, and clay and mineral scaling [6]. Microbial biofouling is one of the major reasons of flux and rejection decline. Feed water parameters, design of the system, and failures in pretreatment systems can contribute to microbial growth [7].

Several autopsy studies exist in [8–14]. In one of the studies, Boubakri and Bouguecha (2008) found that calcium carbonate (CaCO_3_) scaling caused internal obstruction of the cartridge filters. Poor organophosphate based antiscalants interact with aluminum and this interaction caused membrane scaling. According to the autopsy results, they found that stronger antiscalants should be used in the system [8]. Fernandez-Álvarez et al. (2010) conducted a membrane autopsy research on spiral wound membranes which had been in service for 8 years. Conventional pretreatment was used before membrane treatment system. Their research revealed that conventional pretreatment was enough to keep a reverse osmosis (RO) desalination plant in operation for 8 years. In conventional pretreatment, hypochlorite usage was efficient to control biofilm formation; however, it was not efficient enough for the removal of small particles. Membranes were clogged due to quartz, clay, muscovite, and chlorite [9]. Butt et al. (1997) conducted a membrane autopsy research and they found that iron and calcium-alumino-silicates were the major reason for shortening of membrane life. Calcium-alumino-silicates have combined effect on iron and aluminum elements as well as silica compounds [10]. Discart et al. (2014) investigated fouling process of a full-scale ultrafiltration (UF) plant. They studied the role of transparent exopolymer particles (TEPs). They found interactions between iron (Fe) (flocculant), algae, and TEPs. Membrane autopsy showed a thick iron-rich fouling layer on membrane surface. They advised the use of different type of flocculants as well as cleaning agents [11]. Lee and Kim (2012) analyzed local fouling of a hollow fiber membrane submerged in a pilot scale drinking water treatment plant using membrane autopsy. Membranes placed near the aerators had the highest flux recovery after chemical cleaning. Irreversible fouling was faced in samples taken from areas near the open ends of the fibers [12]. Membrane autopsy can also be used to verify differences between plant operation types. For instance, Kim et al. (2008) used membrane autopsy to find out the effectiveness of microfiltration and ultrafiltration pretreatment. Their research revealed that UF pretreatment is more effective than microfiltration (MF) pretreatment, because UF has tighter pores than MF which results in rejection of colloidal foulants much more [13]. Membrane autopsy is not just suitable for filtration processes like UF or RO. It is also a useful tool in understanding fouling mechanism in membrane distillation. Zarebska et al. (2014) applied membrane autopsy to membrane distillation process used for the recovery and concentration of ammonia from mature swine to understand foulant characteristic and foulant mechanism. They found that main foulants are organic matters and the main problem is decrease in surface contact angle after fouling which leads to decrease in surface hydrophobicity and decrease in distillation performance [14].

In this study, an autopsy research was carried out for nanofiltration membranes that were used in Komurcuoda leachate treatment plant, Istanbul, since 2010. Surface aerators used in biological basin were exchanged with bottom diffusers at the last quarter of 2012. After this exchange, MBR plus nanofiltration treatment performance and the amount of color which is removed from leachate decreased. Chemical cleaning was not as effective as before and lack of sufficient chemical cleaning rapid decrease was seen in membrane flux. Although membranes were renewed, rapid membrane flux decline problem could not be prevented. Finally, an autopsy research was performed to put forward the problem about replaced membranes.

## 2. Materials and Methods

### 2.1. Description of Plant

**Figure 1:**
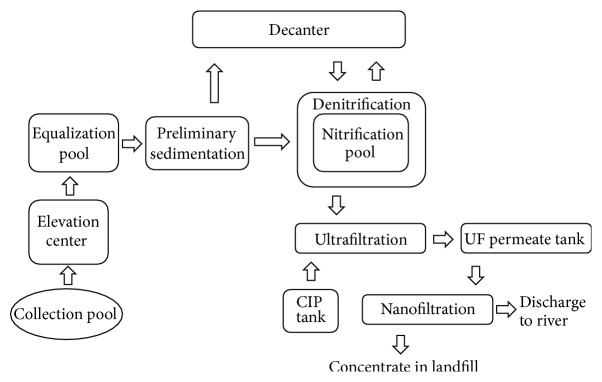
Komurcuoda leachate treatment plant flow diagram.

Istanbul is the biggest city of Turkey having a population of about 17 million. Waste production is about 14,000 tonnes/day and all waste is stored in landfills. Among them, Komurcuoda landfill has the capacity of 5,500 tonnes of waste. For 15 years, totally 17 million tonnes of waste have been stored in Komurcuoda landfill. Komurcuoda leachate treatment plant treats 1,200 m^3^ of leachate/day with membrane systems. High density polyethylene (HDPE) piping is used in drainage system to collect leachate in the plant. A gravel layer is placed above the drainage system. Leachate was transferred to collector basin and then pumped into the equalization tank by using plunger pumps. Leachate treatment plant consists of a primary sedimentation tank, denitrification-nitrification tanks, and UF + NF unit (Figure 1).

### 2.2. Autopsy of Membranes

In this study, visual and physical inspection of the modules, SEM, EDS, ICP, FTIR-ATR, XRD analysis, membrane performance, and dye removal tests were conducted for membrane autopsy. Primarily, module outer shell is examined. Afterwards all the connection areas, gaskets, and antitelescoping devices on the module were examined visually. Meantime, membrane outer shell was cut longitudinally. In order to prevent membrane contamination from particles which are scattered during module outer shell flaking, a vacuum system was used. Finally, each membrane sheet and spacer were examined visually and each of them was designated separately.

### 2.3. SEM/EDS Analysis

SEM provides atomic level analysis and gives crucial information about the membrane surfaces [15]. Membrane surface and spacer samples were characterized by using an FEI Quanta FEG 200 SEM. Wet membrane samples were first frozen in liquid nitrogen and then broken into two parts. Samples were then coated with 5 nm of Palladium and Gold (Pd-Au) by using Quorum SC7620 ion sputtering equipment. SEM images were taken at 30,000x, 8,000x, 3,000x, 600x, and 100x magnifications.

### 2.4. Surface Deposit Analyses

ICP-OES, XRD, and FTIR analyses were carried out for elemental analysis of the surface deposits. DOC analysis was done to find the total organic content of the surface deposits.

In ICP-OES and dissolved organic carbon (DOC) analyses, deposit solution was obtained using several random membrane sheets. Each piece of random membrane sheets was dipped into 100 mL of 0.8 M nitric acid (HNO_3_) or 0.1 M sodium hydroxide (NaOH) solutions and was kept in ultrasonic bath for 5 h to obtain deposit solution. Caustic solution is neutralized by using HNO_3_ before analyses. Perkin Elmer Optima DV 3000 equipment was employed for ICP-OES analysis. Deposit solution was scanned for strontium (Sr), mercury (Hg), lead (Pb), cadmium (Cd), zinc (Zn), nickel (Ni), cupper (Cu), chromium (Cr), iron (Fe), sulphur (S), calcium (Ca), magnesium (Mg), sodium (Na), and aluminium (Al) elements. Shimadzu TOC-VPN equipment was used to find the total organic content. Samples were filtered with 0.45 *μ*m filter prior to analysis. Perkin Elmer Spectrum 100 and Bruker Advance D8 instruments were used for FTIR and XRD analyses, respectively.

### 2.5. Performance and Dye Test

Both magnesium sulphate (MgSO_4_) rejection and dye tests were performed by using dead-end stirred cell system (Sterlitech, HP4750). Dye rejection test was performed to determine the dye rejection capability of the membranes and the presence of defects on membrane surface. The measurements were triplicated with different membrane samples obtained from random membrane sheets. Average flux values were used.

The membranes were placed at the bottom of the stirred cell having an effective filtration area of 14,8 cm^2^. 2000 mg/L MgSO_4_ salt solution was filtered at 5 bars, for 15% recovery at 25°C. Dye tests were conducted at 5 bars by using 10 mg/L reactive orange dye. Magnetic stirrer was used in the cell to obtain cross flow effect on the membrane surface.

## 3. Results and Discussion

### 3.1. Physical Inspection

**Figure 2:**
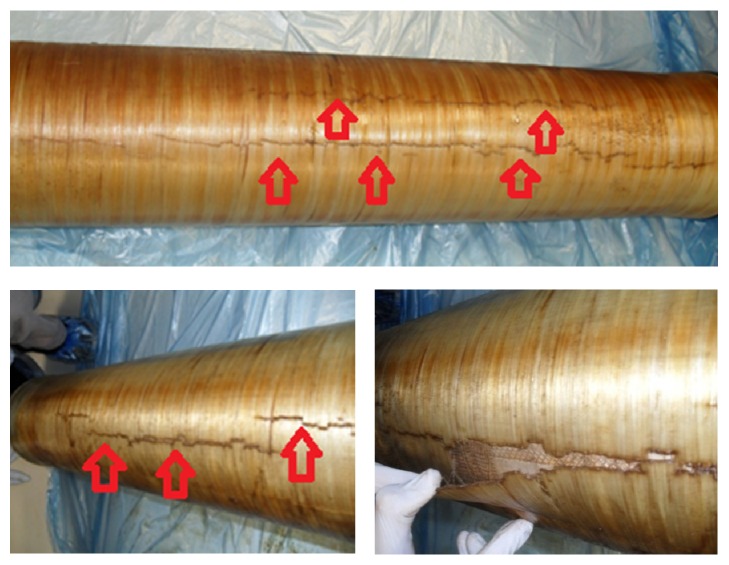
Cracked module shells due to high pressure drop (arrows indicate cracks).

Antitelescoping devices, junction points, and gaskets on the module were intact; however, module outer shell had cracks on it longitudinally as can be seen in Figure 2. This may be related to the high pressure drop between the entrance and exit. Pressure drop originated from fouling may be converted to axial pressure by the module. This axial pressure may be caused by the longitudinal cracks on the module [16]. These cracks led to deterioration of membrane unity and stability. Because of that, membrane sheets can be damaged or flow can be decreased.

**Figure 3:**
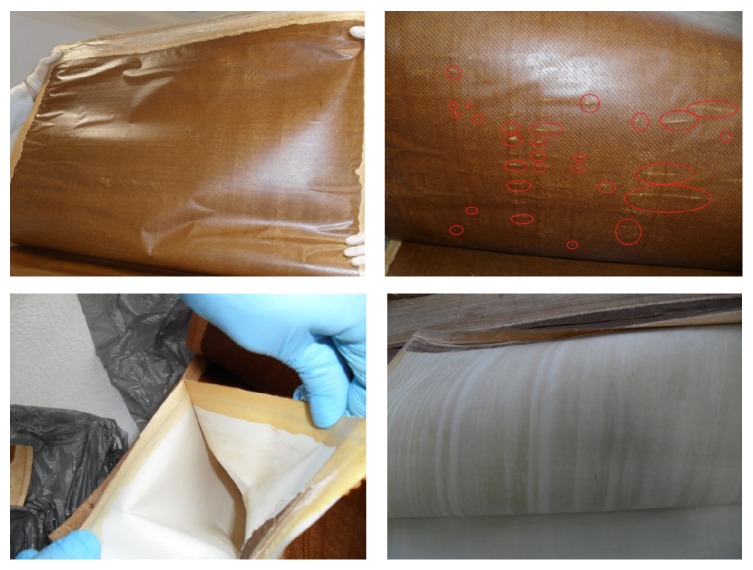
Membrane leaves and permeate spacer images taken during physical inspection (circles indicate surface damage).

After removing of module outer shell, a brownish colored film layer was observed on each membrane sheet. This was the proof of iron fouling and biofouling (Figure 3). After the inspection of each membrane sheet, the defects on the membranes were detected as can be seen in Figure 3. These defects can be due to the combined effects of cracked shell and high pressure which led to unity and stability losses. Since module was no longer intact, applied pressure may have forced spacers to press the membrane surface. Because of that membrane surface and active layer may be deteriorated. Permeate spacers were discarded from the membrane module for observational analyses. It was seen that permeate spacers had also a brownish colored film layer which may result from damage taken (Figure 3).

### 3.2. SEM/EDS Analysis

**Figure 4:**
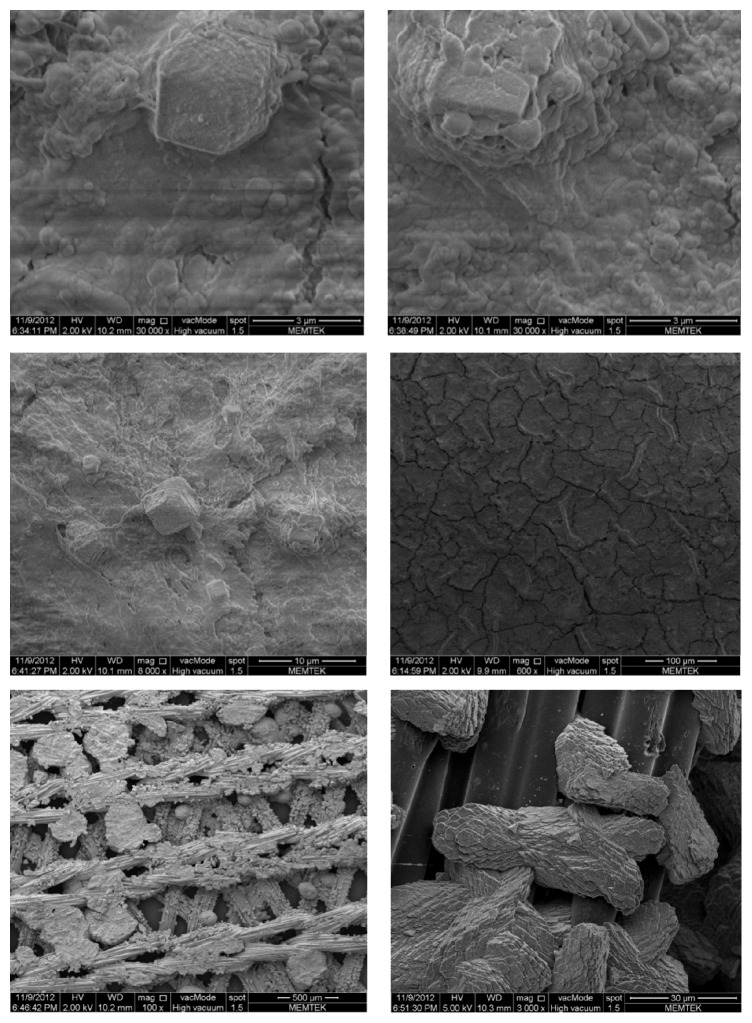
SEM analysis of membranes and spacer.

As seen in Figure 4, significant amount of organic matters was deposited on the membrane surface and a biofilm layer can be clearly seen on the membrane surface. Moreover, scaling formation that resulted from inorganic deposits was observed and it was predicted as calcium carbonate (CaCO_3_) scaling based upon the shape of scaling. Chemical washing effectiveness can be affected by this inorganic scalant entrapped within organic layer.

Additionally, SEM analysis of the permeate spacer was conducted. Unexpectedly, significant amount of inorganic scales was found also on permeate spacers. EDS analysis proved that scaling arised from calcium carbonate as well.

**Figure 5:**
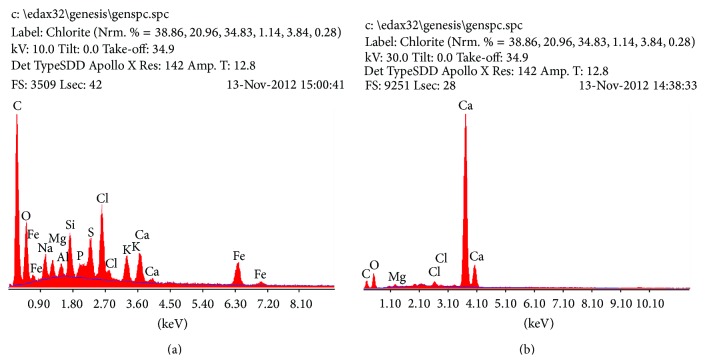
EDS spectrums of membrane (a) and spacer (b) surfaces.

Major foulants found in the membrane and spacer's deposit layer were calcium, chlorine, silicium, magnesium, iron, and sodium according to EDS analysis (Figure 5).

### 3.3. Surface Deposit Analysis

ICP and DOC results were given in Table 1. Acidic solution was used to remove inorganic foulants, whereas caustic solution was used for organic foulants. When NaOH was used, obtained DOC and calcium amount within the solution was higher. From the SEM images, it can be seen that some scalants were covered with biofilm. Probably, most of CaCO_3_ scaling was covered with a biofilm. Biofilm layer removal by caustic solution enhanced the removal of CaCO_3_ better than that of nitric acid. Although sonication was used to enhance the removal rates, caustic solution was not as effective as acidic solution for removing the inorganic foulants. It may be due to the aggregation of divalent (or more) metals which makes complexes with organic matter. Therefore, the effectiveness of caustic solution may be decreased.

According to ICP results, the amount of iron was found as 4.5 g/m^2^. Existence of high amount of iron could deteriorate thin film layer of the membranes. Iron may have catalytic effect in high concentrations, which can promote the oxidation of membrane thin film layer. ICP results also showed the existence of strontium, magnesium, sodium, and calcium in significant amounts. These elements can result in scale formation which can lead to flux decrease.

As mentioned before, the aeration type of the treatment plant was changed. This change brings about rapid flux decline as well as decrease in the rate of color removal. If the oxygen concentration in water increases over 5 mg/L, ferrous (Fe^2+^) iron is converted into ferric (Fe^3+^) iron form. Ferric iron is insoluble colloidal form of iron and it may clog the membrane pores. So the application of bottom diffusers instead of surface aerators changed the concentration of oxygen in wastewater. Because of that, ferric iron formation was enhanced. Besides, no iron antiscalant was used. This enhanced membrane performance loss.

Three different membrane samples were analyzed in XRD. XRD spectrum was given in Table 2. Salt crystals such as calcium sulfate, silica dioxide, barium sulfate, and illite clay were found in XRD spectrum.

Illite clay exists generally in soil and it can mix with water rapidly. XRD detected this mineral probably because of the existence of soil in wastewater. When certain minerals form scaling, they adsorb some elements into their structures. For example, during CaCO_3_ scaling, magnesium molecules penetrated into scales or during barium sulfate scaling, lead element penetrated into the scale structure which worsened crystallization.

**Figure 6:**
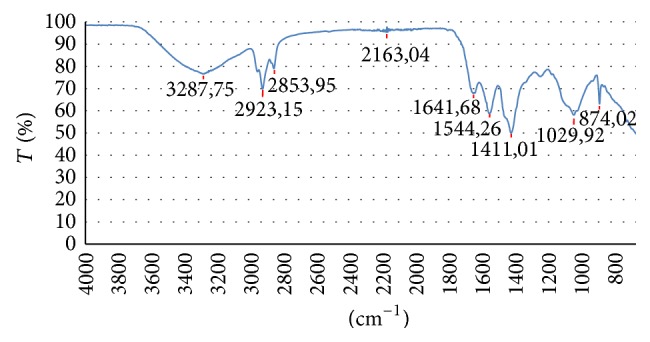
ATR-FTIR spectra of fouled membrane surface.

FT-IR spectra of the fouled membrane surfaces were given in Figure 6. Broad absorption peak between 3200 and 3400 cm^−1^ indicated the presence of polysaccharides as foulants [17]. The peaks around 1640 and 1540 cm^−1^ showed amide I and amide II bonds which show protein presence [18]. The peaks around 1030 cm^−1^ belonged to the C-O bonds which can be an indicator of carbohydrates. FT-IR results validated biofilm layer existence on the membrane surface.

### 3.4. Performance and Dye Rejection Test

Performance and dye rejection test results were given in Table 3. Average pure water flux of randomly selected membranes was found as 51.9 L/m^2^·h. During rejection tests, the average flux was found as 12.8 L/m^2^·h. Due to technical specifications, magnesium removal can be up to 97%. However, performance test results indicated 54.1% rejection obtained at 2,000 mg/L MgSO_4_ concentration at 5-bar pressure.

**Figure 7:**
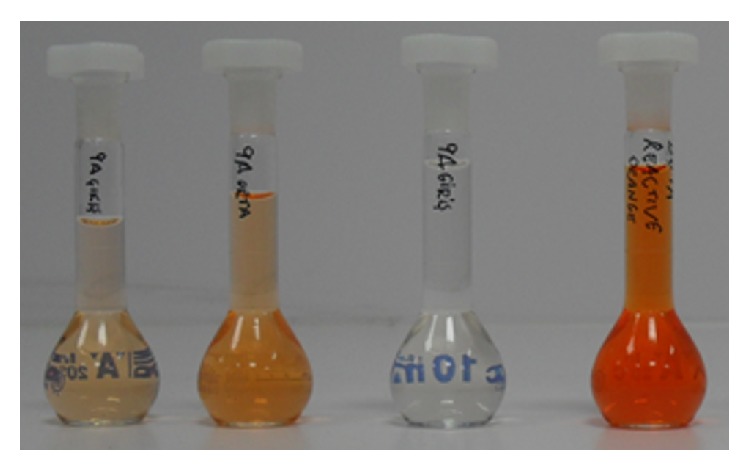
Feed dye solutions (right) and permeates collected using membranes taken from three different parts from membrane.

Dye rejection tests were conducted by using 10 mg/L reactive orange dye solution. Three random membrane samples were used in finor dye rejection test. This test aimed to find whether membranes are capable of removing color or not. Therefore, the rate of color removal was not studied in details. After dye filtration, color existed within permeates which indicated poor color rejection. Color existence in permeate can be associated with the damaged active layer on membrane surface. Different permeates obtained during dye filtration were presented in Figure 7.

### 3.5. Problem Diagnosis and Strategies for Increasing Treatment Efficiency

Membrane autopsy revealed that exchanging surface aerators with bottom diffusers increased the oxygen concentration in tank. Increased oxygen amount increased ferric iron formation (insoluble form). Particulate iron caused rapid fouling of the membranes. Also particulate iron may enhance the oxidation of thin film composite (TFC) surface. Loss in the rejection performance of the membranes may occur due to TFC layer oxidation. When membrane rapidly fouled, pressure dropped so permeate amount decreased. Plant operators increased the pressure applied to compensate the permeate rate. However, pressure increase damaged outer shell of membrane module.

One of the strategies suggested for increasing the treatment efficiency was the usage of more effective antiscalant against iron. Antiscalant specifications were given in Table 4. In the treatment plant, Vitec 3000 was used as scalant. Suggested antiscalant was Vitec 400, which is more effective for iron and silica scaling.

**Figure 8:**
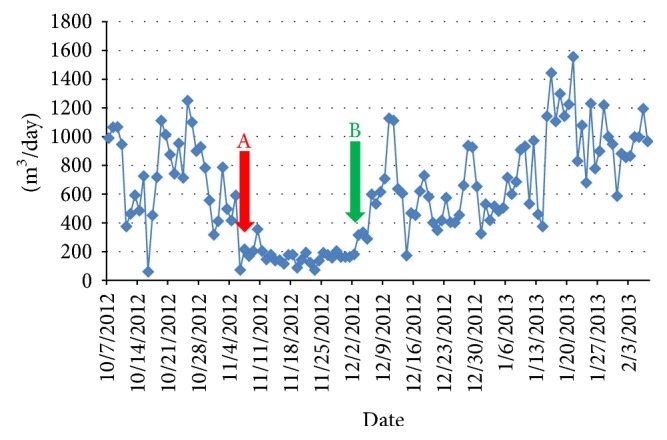
Flow rate graphic of NF membrane plant (A: when problem started and B: when problem solved).

Performance losses and performance enhancements of the treatment can be clearly seen in Figure 8. In Figure 8, point “A” indicated starting point of the problem whereas point “B” indicated the point where the problem was solved. After the membrane autopsy, and proper problem diagnosis, treatment plant capacity increased.

## 4. Conclusion

Nanofiltration membrane module sample which was obtained from Komurcuoda leachate treatment plant was used in this study. Foulant analyses and membrane failure diagnosis were conducted. According to the results, the following conclusions may be drawn.

- Membrane fouling caused pressure drop which caused damage to membrane outer shell and decreased overall system performance. It is important to measure pressure between module inlet and outlet.
- Rapid fouling and flux decline may result from the operational conditions. As flux declines, pressure should be controlled. When pressure increases, more persistent fouling layer covers the membrane surface. Effective chemical cleaning is needed. Pressure increase can be good to solve the problem for short term; however, for long term, effective cleaning should be considered.
- Brownish deposit layer and ICP-OES analyses showed high iron concentration. Changing the type of aeration within aeration basin indirectly increased iron content.
- The presence of iron can oxidize polyamide thin film layer which causes membrane rejection performance loss. Also, cracked module may damage the membrane surface.
- After the usage of iron-effective antiscalants, treatment plant performance returned to its initial point.

## Figures and Tables

**Table 1:** ICP-OES and DOC results of extracted surface deposition in the presence of HNO_3_ and NaOH.

0,8 M HNO_3_			0,1 M NaOH		
Element	mg/L	mg/m^2^	Element	mg/L	mg/m^2^
Sr	7,88	105,07	Sr	0,001	0,01
Hg	0,0007	0,01	Hg	0,0007	0,01
Pb	0,02	0,27	Pb	0,02	0,27
Cd	0,001	0,01	Cd	0,001	0,01
Zn	0,02	0,27	Zn	0,02	0,27
Ni	0,008	0,11	Ni	0,008	0,11
Cu	0,01	0,13	Cu	0,01	0,13
Cr	0,004	0,05	Cr	0,004	0,05
Fe	335,1	4468	Fe	0,855	11,4
S	0,001	0,01	S	0,001	0,01
Ca	32,15	428,67	Ca	128,8	1717,33
Mg	84,45	1126	Mg	12,29	163,87
Na	1294	17253,33	Na	—	—
Al	2,28	30,4	Al	2,537	33,83
DOC	25,49	—	DOC	40,98	—

**Table 2:** Matters observed on surface using XRD analysis.

Scaled matters	Formula
Calcium sulfate (anhydrite)	(CaSO_4_)
Calcium carbonate with magnesium	(Ca_0,9_Mg_0,1_(CO_3_))
Calcium carbonate (aragonite)	(CaCO3)
Silicon dioxide (quartz)	(SiO_2_)
Barium sulphate with lead	(Ba_0,9_Pb_0,1_(SO_4_))
Sodium chloride (halite)	(NaCl)
Illite clay	(KAl_2_(Si_3_AlO_10_)(OH)_2_)
Kaolinite	(Al_2_Si_2_O_5_(OH)_4_)
Strontium carbonate (strontianite)	(SrCO_3_)

**Table 3:** Flux and rejection values of membrane.

	Sample 1	Sample 2	Sample 3	Average value
Flux with pure water (L/m^2^·h)^*∗*^	69.2	62.5	24.2	51.9
Rejection (%)	49.6	57.4	55.4	54.1
Flux during rejection	5.35	21.48	11.55	12.8

^*∗*^12 bars.

**Table 4:** Information of antiscalants.

Product name	Scale inhibitor						Iron dispersant	Colloid and silt dispersant	Silica inhibitor
	CaCO_3_	CaSO_4_	SrSO_4_	CaPO_4_	BaSO_4_	MgOH			
Vitec 3000	+	+	+		+			+	

Vitec 4000	+	+	+		+		+	+	+
